# INTS8 is a therapeutic target for intrahepatic cholangiocarcinoma via the integration of bioinformatics analysis and experimental validation

**DOI:** 10.1038/s41598-021-03017-0

**Published:** 2021-12-08

**Authors:** Qi Zhou, Li Ji, Xueying Shi, Dawei Deng, Fangyue Guo, Zhengpeng Wang, Wenhui Liu, Jinnan Zhang, Shilin Xia, Dong Shang

**Affiliations:** 1 Clinical Laboratory of Integrative Medicine, The First Affiliated Hospital of Dalian Medical University, No.222 Zhongshan Road, Dalian, China; 2 Institute (College) of Integrative Medicine, Dalian Medical University, No.9 West Section Lvshun South Road, Dalian, China; 3 Gastroenterology Department, DongZhiMen Hospital, Beijing University of Chinese Medicine, No. 5 Haiyuncang, Dongcheng District, Beijing, China; 4 Department of General Surgery, The First Affiliated Hospital of Dalian Medical University, No.222 Zhongshan Road, Dalian, China

**Keywords:** Tumour biomarkers, Cancer, Biomarkers, Diseases, Risk factors

## Abstract

Intrahepatic cholangiocarcinoma (CHOL) remains a rare malignancy, ranking as the leading lethal primary liver cancer worldwide. However, the biological functions of integrator complex subunit 8 (INTS8) in CHOL remain unknown. Thus, this research aimed to explore the potential role of INTS8 as a novel diagnostic or therapeutic target in CHOL. Differentially expressed genes (DEGs) in two Gene Expression Omnibus (GEO) datasets were obtained by the “RRA” package in R software. The “maftools” package was used to visualize the CHOL mutation data from The Cancer Genome Atlas (TCGA) database. The expression of INTS8 was detected by performing quantitative reverse transcription-PCR (qRT-PCR) and immunohistochemistry in cell lines and human samples. The association between subtypes of tumour-infiltrating immune cells (TIICs) and INTS8 expression in CHOL was determined by using CIBERSORT tools. We evaluated the correlations between INTS8 expression and mismatch repair (MMR) genes and DNA methyltransferases (DNMTs) in pan-cancer analysis. Finally, the pan-cancer prognostic signature of INTS8 was identified by univariate analysis. We obtained the mutation landscapes of an RRA gene set in CHOL. The expression of INTS8 was upregulated in CHOL cell lines and human CHOL samples. Furthermore, INTS8 expression was closely associated with a distinct landscape of TIICs, MMR genes, and DNMTs in CHOL. In addition, the high INTS8 expression group presented significantly poor outcomes, including overall survival (OS), disease-specific survival (DSS) and disease-free interval (DFI) (p < 0.05) in pan-cancer. INTS8 contributes to the tumorigenesis and progression of CHOL. Our study highlights the significant role of INTS8 in CHOL and pan-cancers, providing a valuable molecular target for cancer research.

## Introduction

As a hepatobiliary malignancy subtype, intrahepatic cholangiocarcinoma (CHOL) has attracted increasing attention due to its increasing global incidence and trends^1^, with the largest age-standardized incidence rates increasing in China (average annual percent change: 11.1%) from 1993 to 2012^2^. Moreover, the mortality rate of CHOL shows a rising trend globally and is approximately 1–2/100,000 in most countries^3^. Because of the aggressive and asymptomatic features of CHOL, many patients are diagnosed at an advanced stage. Surgical resection is regarded as the best treatment strategy for achieving a good prognosis and long survival. However, the 5‐year overall survival (OS) rate remains limited to 22–30% after curative hepatectomy due to high recurrence rates^4,5^. Moreover, a 40–80% recurrence rate was reported for CHOL patients after surgical resection^6^. The combination of gemcitabine and cisplatin is regarded as the standard chemotherapy regimen, despite showing limited effectiveness for CHOL. Therefore, it is urgent to improve the sensitivity of diagnosis and effectiveness of targeted therapy for CHOL.

Integrators are transcriptional regulatory complexes comprised of at least 14 subunits^7^. Integrator complex subunit 8 (INTS8) is one of the major components of RNA polymerase II and has been demonstrated to be involved in the cleavage of small nuclear RNAs and transcriptional processes^8,9^. A recent study found that INTS8 was essential for transcription repression, which was induced by recruiting protein phosphatase 2A to prevent transcription elongation and promote transcription termination^10^. A previous study revealed that INTS8 was robustly increased in neurodevelopmental diseases^11^ and numerous tumours^12,13^. Overexpressed INTS8 could facilitate epithelial-to-mesenchymal transition, which is mediated by the TGF-β signalling pathway in hepatocellular carcinoma (HCC)^14^. Increasing evidence has demonstrated that mismatch repair (MMR) genes play an important role in maintaining genomic stability, and DNA methylation regulates gene expression. The loss of key gene functions of MMR genes could induce DNA replication errors, resulting in a high level of somatic mutations. It has been reported that the MMR pathway is potently activated during G1/S phase^15^. DNA methylation is a type of epigenetic modification that can regulate gene expression. As the function of DNA methyltransferases (DNMTs), DNA methylation occurs when the methyl group covalently bonds to the 5′ carbon position of the cytosine in genome CpG dinucleotides^16^. However, studies focused on the role of INTS8 in CHOL are generally lacking.

In the present study, we used the robust rank aggregation (RRA) method to select differentially expressed genes (DEGs) based on the Gene Expression Omnibus (GEO) database. Then, we explored genes at the intersection between DEGs and gene mutation profiles in the CHOL cohort of The Cancer Genome Atlas (TCGA) and identified INTS8 as a candidate gene. We verified the overexpression of INTS8 in CHOL cell lines and human CHOL samples by quantitative reverse transcription-PCR (qRT-PCR) and immunohistochemistry (IHC). Our study showed that high INTS8 expression is closely correlated with poor prognosis across cancers. Moreover, the underlying mechanism may be attributed to tumour-infiltrating immune cells (TIICs), MMR genes, and DNMTs. Therefore, INTS8 was identified as a therapeutic target in CHOL and pan-cancer series; an association was observed between INTS8 expression and TIICs; MMR genes and DNMTs were suggested to mediate INTS8 effects.

## Materials and methods

### Data acquisition and processing

We selected 2 CHOL datasets from the GEO database (http://www.ncbi.nlm.nih.gov/geo/): GSE26566 and GSE32225^17,18^. GSE26566 included 104 CHOL samples and 6 matched surrounding samples. GSE32225 contained 149 CHOL samples and 6 matched surrounding samples. All analyses were undertaken with R version 4.0.4 (https://cran.r-project.org/src/base/R-4/). All expression profiles were downloaded and processed by the “GEOquery” package (www.r-project.org). Considering the differences and batch effects of different platforms, we utilized the “sva” package^19^ to avoid these effects and remove other unwanted variations. In addition, the transcriptome profiles, mutation and methylation data, and clinical information of tumour samples and corresponding samples were obtained from the TCGA database (https://cancergenome.nih.gov/) and analysed by utilizing the “TCGAbiolinks” package^20^. Transcripts per million (TPM) values were applied for subsequent analyses^21^.

### DEGs and RRA analysis

The DEGs were determined between CHOL samples and matched surrounding samples using the “limma” package^22^. RRA was performed for gene list integration^23^, and a score < 0.05 was used to determine the RRA gene set. These data were visualized by a heatmap and volcano plot.

### Identification of INTS8 by mutation analysis and ROC curves

We used the “maftools” package^24^ to visualize the CHOL mutation data. To assess mutated genes present in the RRA gene set, we obtained the intersection of RRA genes and CHOL mutation genes. To evaluate the diagnostic performance of muted RRA genes, receiver operating characteristic (ROC) curves were generated by using the “pROC” package. Next, we selected the optimal efficacy indicators based on the areas under the curve (AUCs) for further research. The patients were stratified into two groups according to the median expression of INTS8. DEGs between the high and low INTS8 groups were confirmed by using the “limma” package. The cut-offs for the DEGs were as follows: |log2 fold change (FC)|> 1 and false discovery rate (FDR) < 0.05. A heat map constructed by using the “ggplot2” package was used to visualize the DEGs.

### Functional enrichment of mutated RRA genes and INTS8-related genes

To identify the possible pathways and biological functions of the 5 mutated RRA genes and differential INTS8-associated genes, we applied the “clusterProfiler” package^25^ to perform Gene Ontology (GO) and Kyoto Encyclopedia of Genes and Genomes (KEGG) analyses. The protein–protein interaction (PPI) network of the 5 mutated RRA genes was constructed via the Search Tool for the Retrieval of Interacting Genes/Proteins (STRING, http://string-db.org/) online database and then visualized by Cytoscape v.3.7.1 (https://cytoscape.org/). Moreover, we used Molecular Complex Detection (MCODE) to explore functional clusters in the PPI network. To further understand the functions and biological pathways related to INTS8, gene set enrichment analysis (GSEA) was performed on the INTS8 gene by utilizing GSEA software (v.3.0).

### CIBERSORT estimation

To characterize the tumour microenvironment, we used “CIBERSORT” (R package) (http://cibersort.stanford.edu/)^26^ to explore the relative proportions and absolute fraction scores of 22 subtypes of TIICs in CHOL tissues. Moreover, the association between INTS8 expression levels and the infiltration of TIICs was assessed and visualized by heatmaps and violin plots.

### Association of INTS8 gene expression with clinical outcome in different tumours

To explore the influence of the expression level of INTS8, we carried out analyses using a publicly available database. We retrieved data from The Cancer Cell Line Encyclopedia (CCLE, https://portals.broadinstitute.org/ccle)^27^ and the Genotype-Tissue Expression (GTEx) project^28^ to investigate the gene expression data of INTS8 in a range of tumour tissues and cell lines. Moreover, we downloaded pan-cancer mutation data from the TCGA database and analysed the mutations of INTS8 in samples of 32 different tumour types. Furthermore, we used Cox regression and the Kaplan–Meier method to evaluate the association of INTS8 gene expression with clinical outcome in different cancers. p < 0.05 was regarded as the cut-off to verify the prognostic role of INTS8.

### Association of INTS8 expression with MMR genes and DNA methylation

To reveal the role of INTS8 in cancer progression, we evaluated the relationship between the expression level of INTS8 and 5 key DNA MMR genes (including MLH1, MSH2, MSH6, PMS2, and EPCAM). In addition, we performed an integrative analysis of DNA methylation and INTS8 expression to determine its underlying mechanism in pan-cancer.

### Real-time PCR

A human normal biliary epithelial cell line (HIBEC) and 3 CHOL cell lines (including HCCC-9810, RBE, and CCLP-1 cells) were used to detect the mRNA expression of INTS8. Total RNA and cDNA synthesis was performed by following the manufacturer’s instructions (Accurate biotechnology, China). Gene expression was measured on an ABI 7500 system by using a SYBR Green kit (Accurate biotechnology, China). The forward primer for INTS8 was 5′-TGCTGGAGGAGTCACTGTTGGAG- 3′, and the reverse primer for INTS8 was 5′-TTATCAGGCGGAGGTTGAACTTGG-3′.

### IHC

A total of 155 paired CHOL and 5 peritumoural tissue samples were obtained for experimental validation. Informed consent was obtained from all participants. The study involving human participants was approved by the Ethics Committee of Shanghai Outdo Biotech Company (No. YB M-05-02) and performed following relevant guidelines and regulations. Formalin-fixed paraffin-embedded tissue samples were examined by incubation with primary antibodies (ab18050, Abcam).

### Ethical approval

Informed consent was obtained from all participants. The study involving human participants was approved by the Ethics Committee of Shanghai Outdo Biotech Company with NO. YB M-05-02, and performed following relevant guidelines and regulations.

## Results

### Identification of robust DEGs in GEO

**Figure 1:**
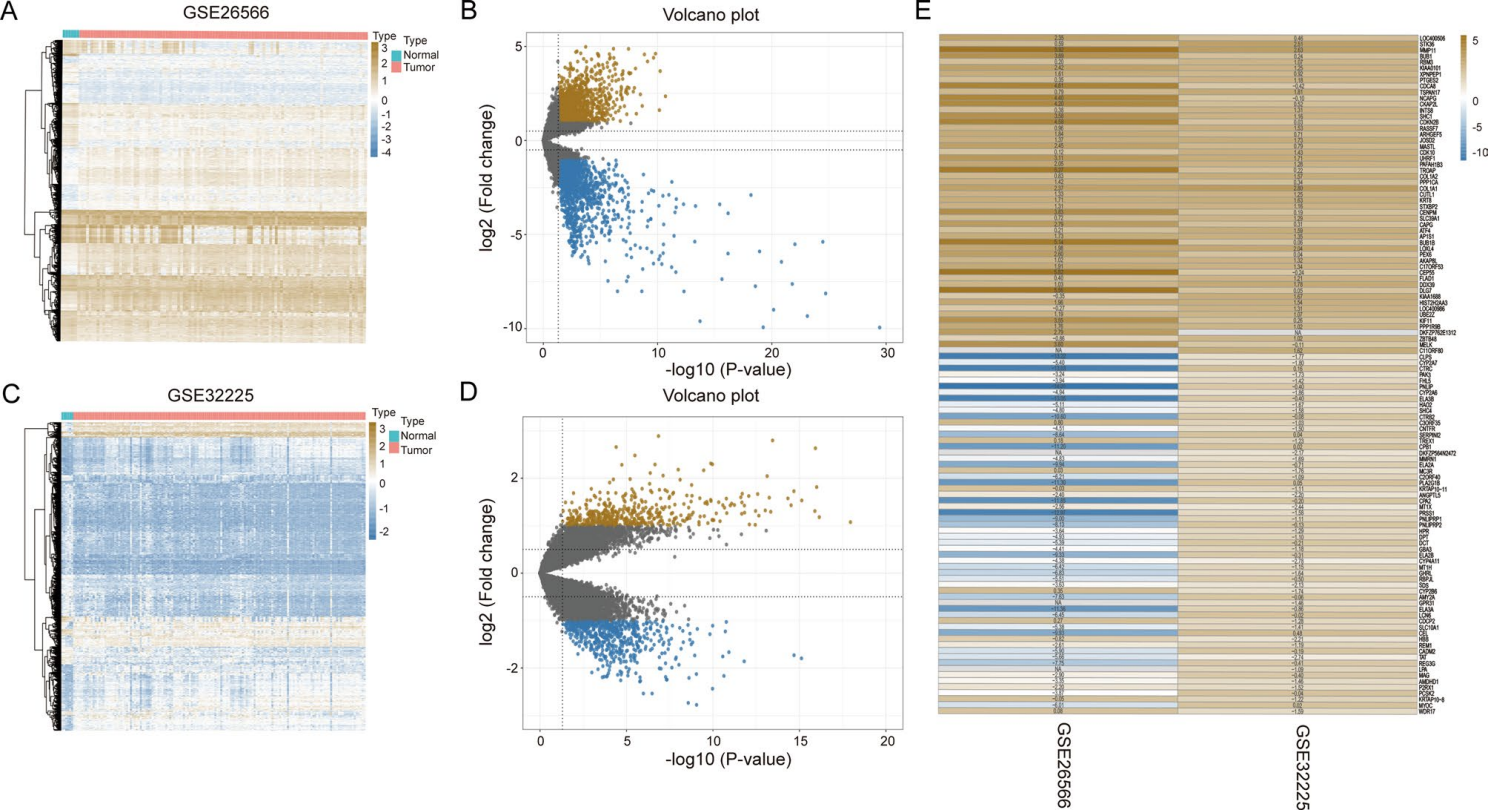
Identification of RRA gene set. (**A**–**D**) Differentially expressed genes of 2 GSE datasets. (**E**) Visualization of the RRA gene set.

Based on the DEG results, a total of 710 significantly upregulated and 903 significantly downregulated DEGs were confirmed in GSE26566, and 432 significantly upregulated DEGs and 566 significantly downregulated DEGs were identified in GSE32225. The DEGs are shown by heatmaps and volcano plots in Fig. 1A–D. Furthermore, these DEGs were integrated by the RRA method with a score < 0.05. Then, the RRA gene set was visualized by a heatmap, as shown in Fig. 1E. As a result, an RRA gene set was obtained for further investigation.

### Functional enrichment and PPI network analyses of the RRA gene set

GO and KEGG enrichment analyses elucidated the functions of the RRA gene set (Supplementary Fig. 1A,B). The RRA gene set was obviously enriched in biological processes, such as lipid catabolic process, digestion, drug catabolic process, and eicosanoid metabolic process. In addition, the RRA gene set participated in pancreatic secretion, fat digestion and absorption, protein digestion and absorption, and focal adhesion (Supplementary Fig. 1C,D). A PPI network of the RRA gene set, which included 202 interactions, was constructed to identify protein interactions and was visualized by Cytoscape (Supplementary Fig. 2A,C). Two functional clusters in the PPI network were extracted, suggesting their central roles in this network (Supplementary Fig. 2B). Our results showed that the RRA gene set was associated with some metabolic pathways.

### Mutation landscape of the RRA gene set in CHOL

**Figure 2:**
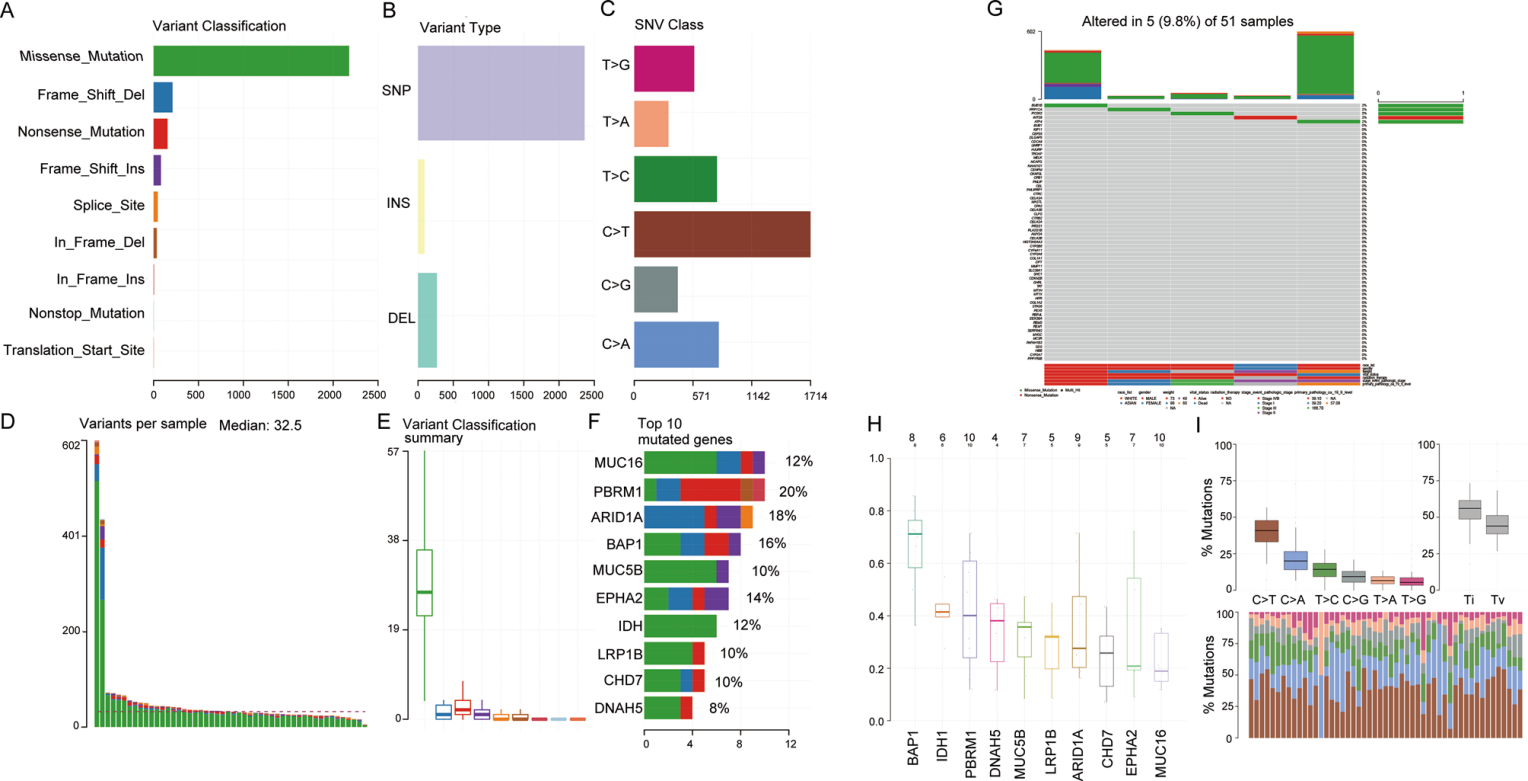
Mutation landscape of the RRA gene set in TCGA-CHOL. (**A**–**C**) According to different classification categories, the classification of mutation types, including missense mutations, SNPs, and C > T mutations, was performed with statistical calculations. (**D**) Total mutation number in each sample. (**E**) Each variant classification in each sample. (**F**) Top 10 mutated genes in TCGA-CHOL. (**G**) The mutation information of 5 mutated genes in the RRA gene set was determined by the waterfall plot. (**H**) The top mutant genes of the RRA gene set are shown by a box plot. (**I**) Mutant base pair ratio of the RRA gene set.

To identify the mutational landscape in CHOL patients, the “maftools” package in R software was used. Missense mutations were the predominant type of mutation in patients with CHOL (Fig. 2A). Single nucleotide polymorphisms had a more frequent occurrence than insertions or deletions (Fig. 2B). In particular, C > T remained the most common mutation type of single nucleotide variants in CHOL (Fig. 2C). The mutation types in CHOL are displayed in Fig. 2D,E. The top 10 mutated genes present in CHOL with ranked percentages are as follows: MUC16 (12%), PBRM1 (20%), ARID1A (18%), BAP1 (16%), MUC5B (10%), EPHA2 (14%), IDH1 (12%), LRP1B (10%), CHD7 (10%), and DNAH5 (8%) (Fig. 2F). A total of 5 mutated genes in the RRA gene set were found in mutation profiles, and the mutation information of the RRA gene set was obtained by a waterfall plot (Fig. 2G). BAP1, IDH1 and PBRM1 were the top 3 mutant genes of the RRA gene set (Fig. 2H). The mutant base pair ratio of the RRA gene set showed that C > T was the most common single nucleotide variant in the RRA gene set (Fig. 2I).

### Identification of DEGs between the high and low INTS8 expression groups

**Figure 3:**
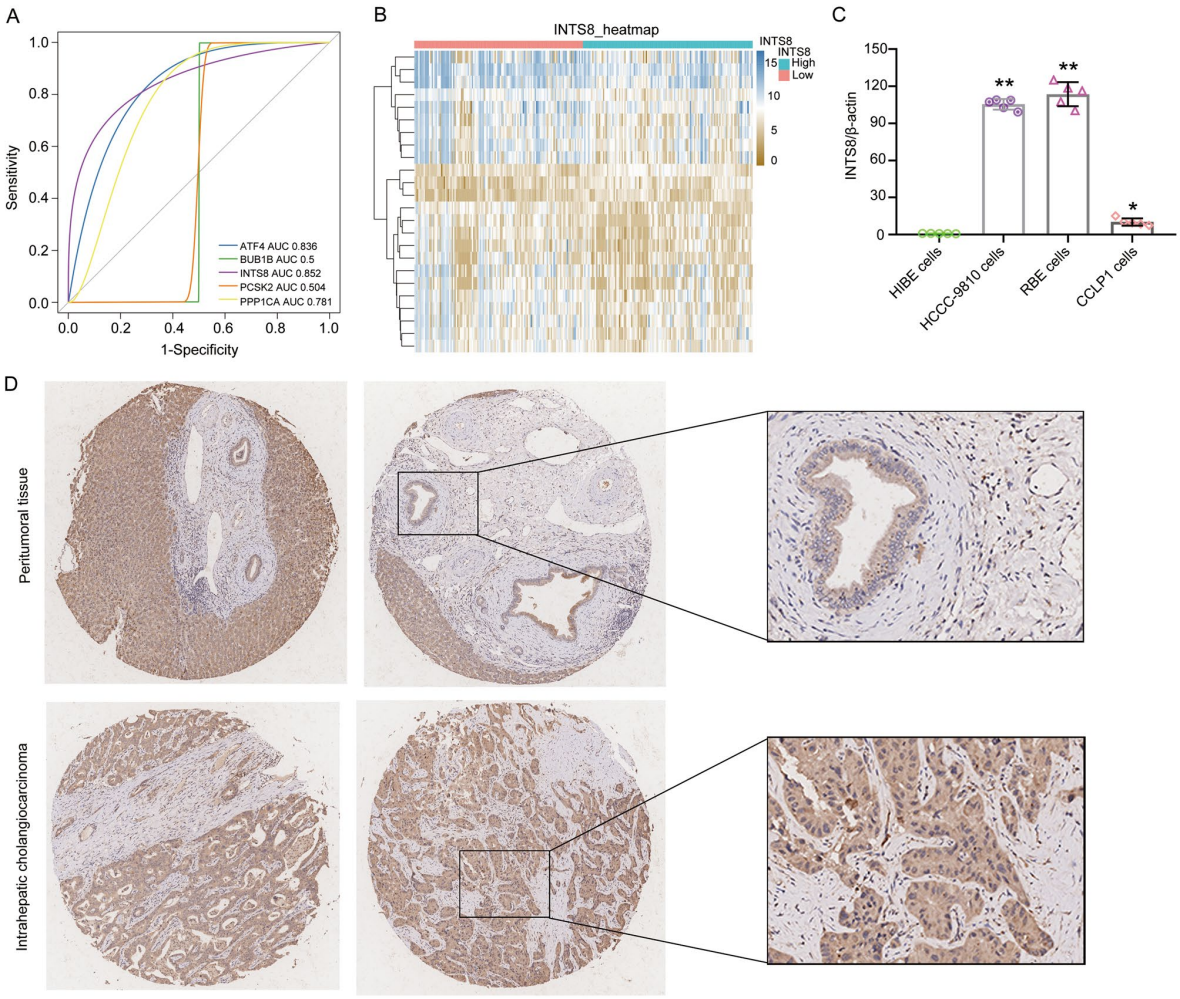
Identification of INTS8 as a candidate gene. (**A**) ROC curves of 5 genes for diagnostic value. (**B**) DEGs in the high and low INTS8 expression groups. (**C**) Expression of INTS8 in HIBEC and 3 CHOL cell lines (including HCCC-9810, RBE, and CCLP-1 cells) by using PCR. (**D**) Representative images of INTS8 IHC staining in human CHOL and adjacent normal tissues.

ROC analysis was applied to determine the diagnostic efficacy of the 5 mutated genes of the RRA gene set. INTS8 had the highest AUC value (AUC = 0.852), followed by ATF4 (AUC = 0.836), PPP1CA (AUC = 0.781), PCSK2 (AUC = 0.504) and BUB1B (AUC = 0.5) (Fig. 3A). Considering that INTS8 had the highest AUC, it was selected as the target gene for further analysis. To explore the underlying mechanism of INTS8 in CHOL, the patients were divided into two groups according to the median expression value of INTS8. DEGs between the high and low INTS8 expression groups were identified (Fig. 3B). Furthermore, we found that the mRNA expression of INTS8 was upregulated in 3 CHOL cell lines compared with HIBE in vitro (Fig. 3C). The protein levels of INTS8 via IHC were also verified to be obviously increased in CHOL patient tissue samples compared with normal tissue samples (Fig. 3D). The experimental results were consistent with those of the bioinformatic analysis.

### Functional enrichment of INTS8 in CHOL

**Figure 4:**
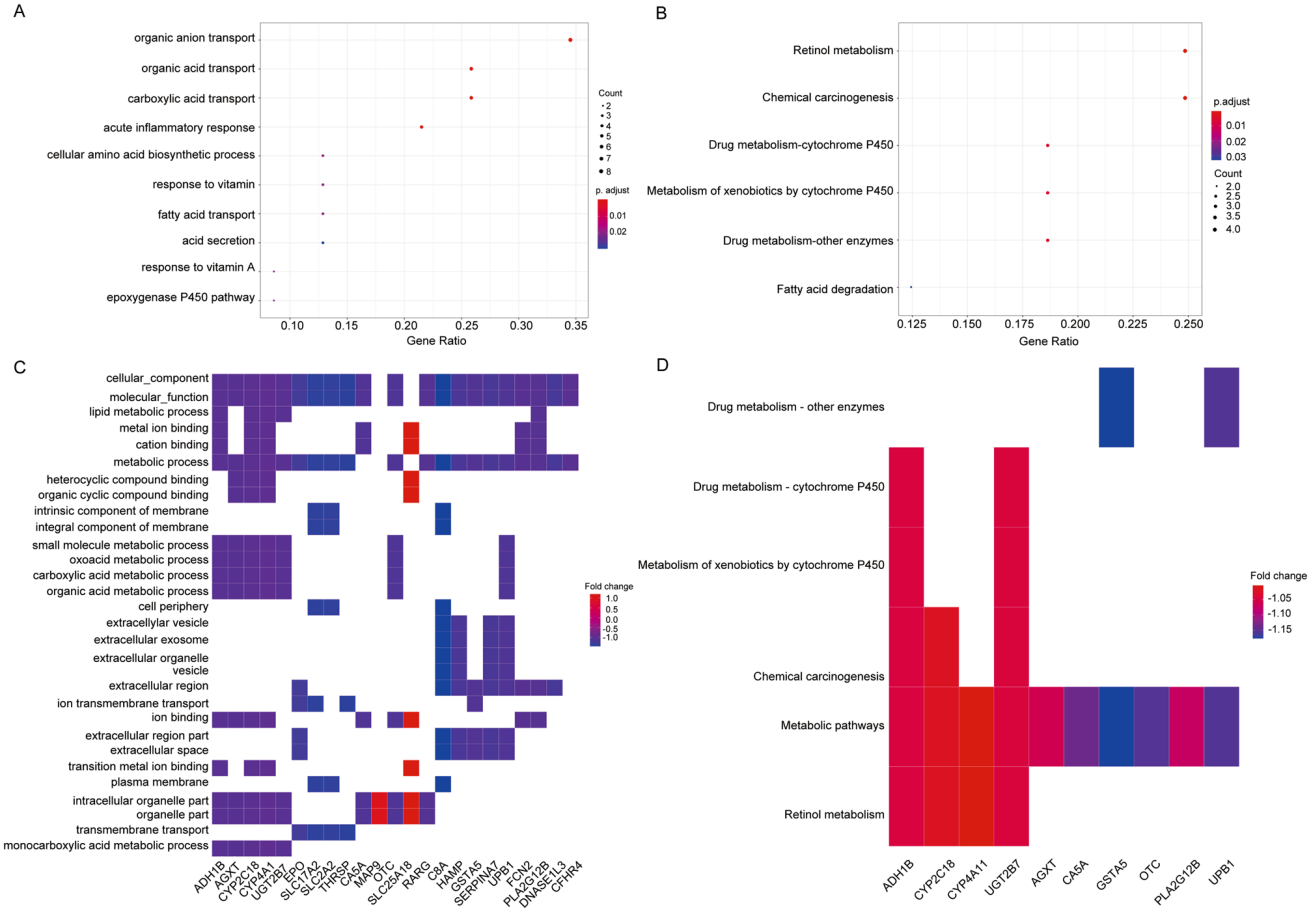
Functional enrichment of INTS8-related genes in CHOL. (**A**,**B**) GO and KEGG analyses of INTS8-related genes. (**C**,**D**) GSEA-GO and GSEA-KEGG analyses of INTS8-related genes.

To identify the biological functions and key candidate pathways of the INTS8-related genes, we performed GO and KEGG analyses. The top 10 GO terms are shown in Fig. 4A. Drug metabolism-cytochrome P450 (CYP), retinol metabolism, chemical carcinogenesis, metabolism of xenobiotics by CYP, drug metabolism-other enzymes, and fatty acid degradation were the most significantly enriched in CHOL patients with high INTS8 expression compared with those with low INTS8 expression (Fig. 4B). To elucidate the molecular mechanisms of INTS8, INTS8-related signalling pathways were analysed by GSEA-KEGG and GSEA-GO (Fig. 4C,D). The results suggested that INTS8 might be related to metabolic pathways, such as CYP and retinol metabolism.

### Association between TIICs and INTS8 expression in CHOL

**Figure 5:**
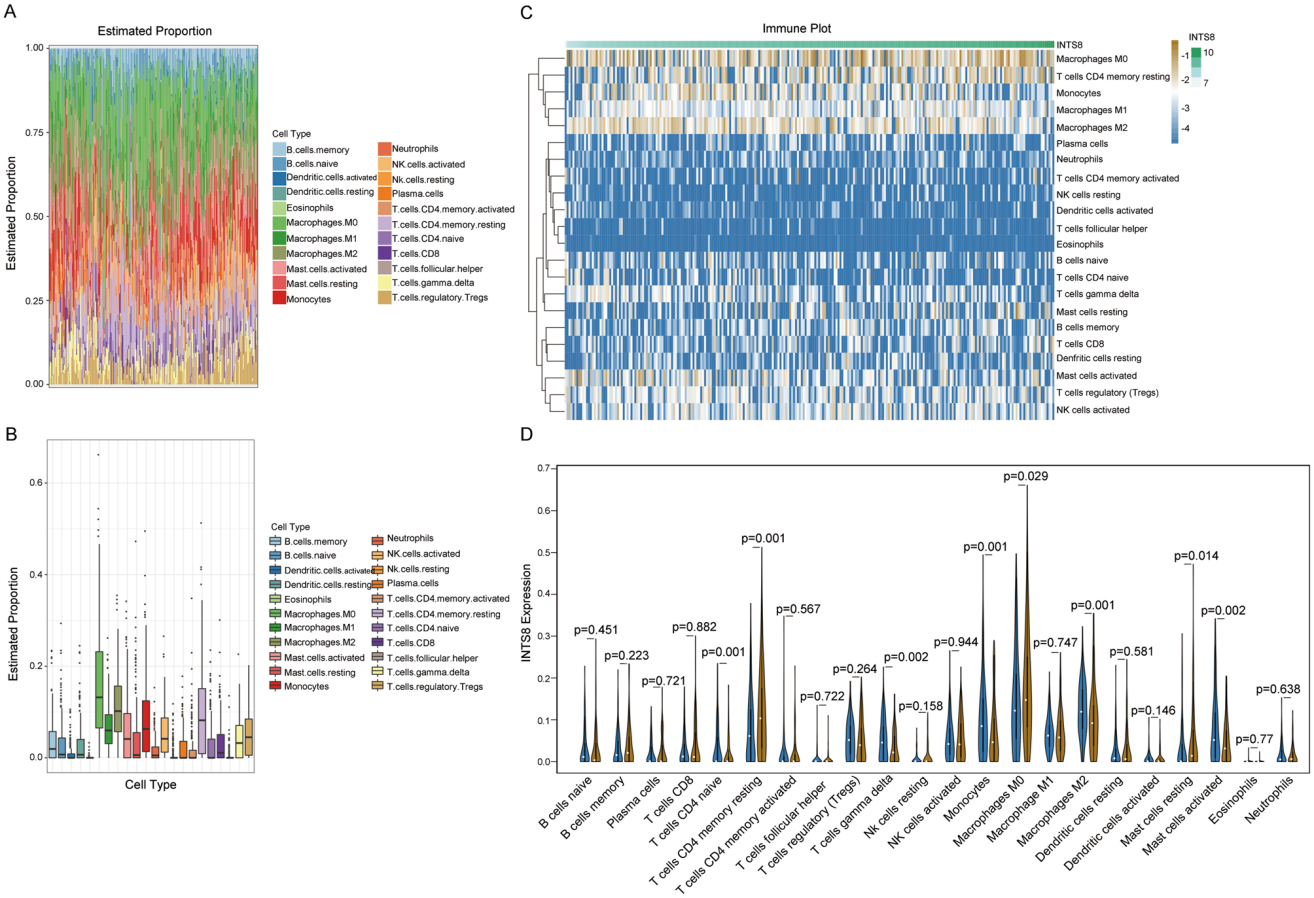
Identification of complex associations between 22 TIIC subsets and INTS8 expression in CHOL. (**A**) Relative proportions of 22 subtypes of tumour-infiltrating immune cells for each sample in CHOL. (**B**) Relative proportions of 22 subtypes of tumour-infiltrating immune cells for each sample. (**C**,**D**) Comparison of the immune cell fraction difference between the low and high INTS8 expression groups. Note: Blue refers to low INTS8 expression, and brown refers to high INTS8 expression.

TIICs significantly impact the development and progression of many types of cancers, including CHOL. By applying CIBERSORT tools, we observed a high level of M0 macrophages, M2 macrophages, monocytes, and resting CD4+ memory T cells and a lower level of activated dendritic cells, eosinophils, neutrophils and activated CD4+ memory T cells in CHOL (Fig. 5A,B). Moreover, we assessed the relationship between TIICs and INTS8 expression in CHOL. We found that the high INTS8 expression group presented a unique TIIC landscape, including a significantly high level of M0 macrophages but a low level of M2 macrophages, an elevated level of resting CD4+ memory T cells but a low level of CD4 naive T cells, and an increased level of resting mast cells but a low level of activated mast cells. In addition, low expression of gamma delta T cells and monocytes was also found in the high INTS8 expression group (Fig. 5C,D).

### INTS8 expression in multiple dimensions

**Figure 6:**
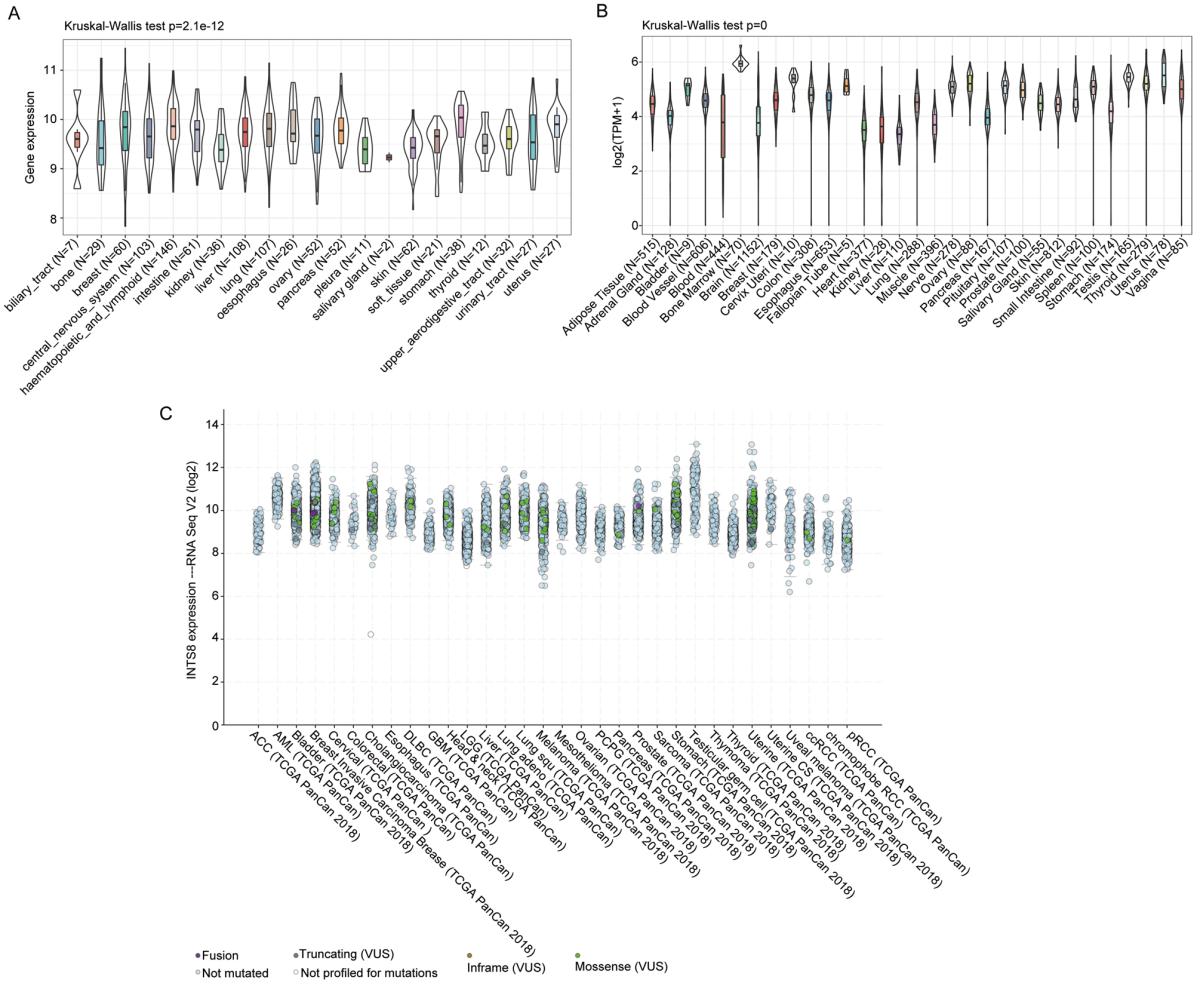
INTS8 expression in multiple dimensions. Expression analysis of the INTS8 gene in CCLE (**A**) and GTEx (**B**) and the somatic mutation profile of the INTS8 gene in the TCGA database (**C**).

Considering the extensive mutational heterogeneity of cancers, we systematically performed large-scale profiling of INTS8 expression in 21 cell lines and 31 related tissues based on CCLE and GTEx. As shown in Fig. 6A,B, the expression levels of INTS8 in diverse cancer tissues, including the biliary tract, liver, and bone marrow, and cell lines were elevated to differing degrees. In addition, we found that INTS8 harboured the most prevalent mutations, such as missense, truncating and fusion mutations, in different tumours (Fig. 6C).

### Associations between INTS8 and clinicopathologic characteristics and survival information

As shown in Table 1, increased INTS8 expression was directly associated with age and grade. INTS8 expression gradually increased from stages I/II to stage IV CHOL. To assess the prognostic capacity of INTS8, we constructed Kaplan–Meier curves for OS, disease-specific survival (DSS), and disease-free interval (DFI) by using multivariate Cox regression analysis. Regarding prognostic outcomes, patients in the high INTS8 group exhibited poor OS, DSS and DFI (p < 0.05) in a pan-cancer analysis (Supplementary Figs. 3–5). These findings suggested that INTS8 expression is a potent potential prognostic biomarker for various cancers.

**Table 1:** Correlation between INTS8 expression and clinicopathological characteristics.

	Variables	INTS8 expression		Total	χ^2^	p value
		Low	High			
Age (year)					4.123	0.042
	< 60	36 (57.14%)	27 (42.86%)	63		
	≥ 60	36 (40.45%)	53 (59.55%)	89		
Sex					0.553	0.457
	Female	30	29	59		
	Male	42	52	94		
Grade					15.516	0.000
	I/II	60 (57.14%)	45 (42.86%)	105		
	III	11 (22.9%)	37 (77.1%)	48		
T stage					0.048	0.773
	T1	1	2	3		
	T2/T4	13	18	31		
N stage					0.192	0.661
	N0	20	23	40		
	N1	12	11	23		
M stage					0.045	0.833
	M0	69	78	147		
	M1	3	4	7		
TNM stage					0.026	0.871
	Ι/II	10	11	21		
	IV	13	13	26		
Tumour size (cm)					0.383	0.536
	≥ 7	33	42	75		
	< 7	32	33	65		

A p-value of < 0.05 was considered as statistically significant.

### MMR genes and DNA methylation genes involved in CHOL

**Figure 7:**
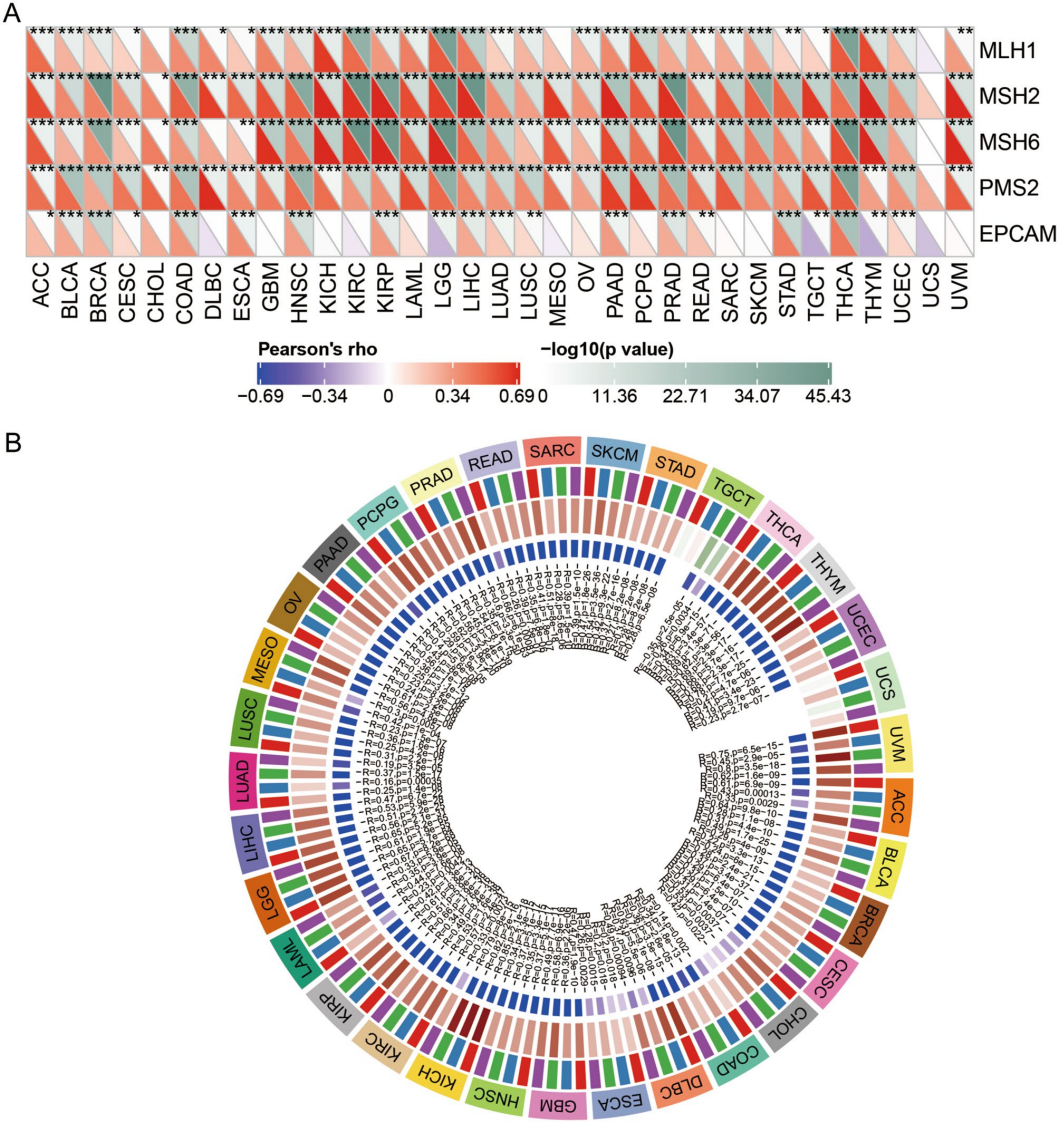
MMR genes and DNA methylation genes involved in CHOL. (**A**) A total of 3 MMR genes were detected in CHOL. The right-angled triangle in the lower left corner represents the Pearson’s correlation between INTS8 and MMR gene expression. The right-angled triangle in the upper right corner represents the p value; *p < 0.05, **p < 0.01 and ***p < 0.001 show significant differences. (**B**) Circle diagram showing the association between INTS8 and DNMTs. The first circle from outer to inner is the abbreviation of cancers. The second circle is the specific DNMTs, with DNMT1 (red), DNMT2 (blue), DNMT3A (green), and DNMT3B (purple). The third circle is the correlation coefficient. Green represents negative correlations; Red represents positive correlations. The fourth circle is the p value. And the fifth circle is the specific value of the correlation coefficient and p.

To explore the underlying DNA repair mechanism associated with INTS8 mutation, we investigated the association between INTS8 and MMR genes (including MLH1, MSH2, MSH6, PMS2, and EPCAM). We found that INTS8 was positively correlated with the expression of MSH2, MSH6, and PMS2 but showed no association with MLH1 and EPCAM. Due to the extensive function of MMR genes in cancers, we performed a pan-cancer analysis to analyse the relationship between INTS8 and MMR genes. Interestingly, a positive association between INTS8 and MMR genes was present in numerous cancers, such as brain lower-grade glioma, liver HCC, and pancreatic cancer (Fig. 7A).

As shown in Fig. 7B, an epigenetic signature was discovered and showed a high correlation between INTS8 and DNMTs (DNMT1: r = 0.31, p < 0.05; DNMT2: r = 0.53, p < 0.05; DNMT3A: r = 0.53, p < 0.05; DNMT3B: r = 0.42, p < 0.05). Furthermore, a pan-cancer analysis of DNMTs was performed and showed that INTS8 was positively related to the expression profiles of 4 DNMTs in most cancers except testicular germ cell tumours. All these results indicated that MMR genes and certain DNMTs may play an important role in INTS8 mutations in CHOL.

## Discussion

CHOL is an extremely aggressive biliary neoplasm with increasing incidence and poor prognosis worldwide^29^. Currently, prognostic model in biliary tract cancers has reached interesting results. For example, the PECS index was identified as a replicable and promising tool to assess the prognosis of biliary tract cancer patients in future clinical practice; it is based on a real-life population and has robust numerosity, with C-indexes of 0.73–0.83 and survival curves showing clear separation. With an integration with clinicopathological model, the potential value of molecular data could contribute to the clinical practice^30^. In this study, the TCGA and GEO databases were applied to systematically analyse the mutational status of RRA genes in CHOL, and 5 mutant genes were found by intersection analysis. Based on the diagnostic efficacy of the 5 mutant genes, we selected INTS8, which had the largest AUC value, for follow-up research, which showed that INTS8 played a significant role in CHOL and even across all cancers.

Various studies have suggested that the integrator complex plays an essential role in RNA processing and transcription regulation. Previous studies have shown that INTS8 mutation can induce severe neurodevelopmental syndrome^11^ and pan-cancer^31^. In this study, we found that INTS8 was significantly overexpressed in CHOL compared to normal samples, which was consistent with the results of IHC and PCR. Our results showed that INTS8 overexpression was positively related to poor prognosis in many tumour types.

The GO enrichment analyses showed that high INTS8 expression was mainly associated with organic anion transport, organic acid transport, carboxylic acid transport and acute inflammatory response. In addition, retinol metabolism, chemical carcinogenesis, drug metabolism-CYP, metabolism of xenobiotics, drug metabolism-other enzymes, and fatty acid degradation were most significantly enriched in CHOL patients with high INTS8 expression compared with those with low INTS8 expression. Retinol is a fat-soluble nutrient that is essential for maintaining physiological functions in many tissues^32^. Retinol metabolism abnormalities caused by genetic or environmental factors could induce developmental pathologies, including mammalian placental and embryonic development^33^, ovary disease^32^ and fatty liver disease^34^. A previous study showed that the administration of retinol facilitated hepatocarcinogenesis development during its early stages^35^. Drug metabolism-CYP was related to DNA methylation-driven genes in prostate adenocarcinoma^36^. In addition, previous data showed that hepatic CYP family enzymes, especially increased CYP2A6 and diminished CYP2E1, might participate in the progression of CHOL^37^. Lipid metabolism is newly recognized as a hallmark of cancer, and inhibiting fatty acid availability could control the development of malignancy^38,39^. Li et al*.* found that CHOL tumorigenesis was insensitive to fatty acid synthase deprivation, which contributed to high fatty acid uptake and resulted in rapid tumour growth. Therefore, promoting fatty acid degradation may be a novel therapeutic approach for CHOL^40^.

DNA damage and repair provide protection for mutation avoidance, which plays central roles in maintaining genome stability^41,42^. To date, it has been reported that 4 major DNA repair pathways are involved in maintaining gene expression, including nucleotide excision repair, base excision repair, MMR, and double-strand break repair^43^. The expression of INTS8 was positively correlated with MSH2, MSH6, and PMS2 but not associated with MLH1 and EPCAM. The IHC analysis^44^ results showed that there was no loss of the expression of DNA repair enzymes/MMR proteins (MLH1, MSH2, PMS2, and MSH6) in either occupational CHOL^45^ or cohorts with CHOL^46^.

MMR gene mutations and tumour MLH1 promoter methylation are the main causes of microsatellite instability (MSI) in patients with colorectal cancer (CRC)^47^. Although the overall number of MSI-high (MSI-H) CHOL cases is low (1.3%), MSI testing of cholangiocarcinoma exhibited an atypical histomorphology, especially in younger patients^48^. EPCAM, a stemness-related marker, is positively correlated with poor prognosis in CHOL and HCC^49,50^. However, we did not observe an association between INTS8 and EPCAM in CHOL.

Recently, epigenetic alterations have been characterized by any heritable modification of chromatin DNA or histone proteins but without changes in the DNA sequence^51,52^; they can be observed in many human cancers and cooperate with genetic alterations to dominate the formation of cancers^53^. DNA methylation is one of the main epigenetic changes and is specifically mediated by the DNMT family (including DNMT1, DNMT1, DNMT3A and DNMT3B)^54^. DNMTs could establish and maintain DNA methylation patterns, which induce gene silencing, transcriptional activation and posttranscriptional regulation mediated by DNMT2-dependent RNA methylation. Here, we found that INTS8 is positively associated with DNMTs in CHOL, suggesting that the effect of INTS8 on CHOL development may be caused by mutations in DNMT genes. Thus, we hypothesized that functional impairment of INTS8, which is associated with MMR genes and DNMTs, promotes malignancy across cancers, suggesting the potential of INTS8 for cancer research.

A significant increase in macrophages was shown in locally advanced CHOL patients compared to metastatic CHOL patients^55^. In our study, the immune landscape showed a distinctly high expression of macrophages in CHOL, which was consistent with the findings of other studies. However, the results showed that low M2 macrophage levels but no significant alteration in M1 macrophages appeared in the high INTS8 expression group, suggesting that remarkable cellular heterogeneity exists in macrophage subtypes. M1 macrophages are currently known to promote inflammation, while M2 macrophages are characterized by anti-inflammatory functions. Thus, whether INTS8 is involved in locally advanced CHOL remains to be experimentally validated. In particular, a case study discovered that gamma delta T cell-based immunotherapy showed no adverse effects and could positively regulate peripheral immune functions in patients with CHOL^56^. We found that a low level of gamma delta T cells was present in the high INTS8 expression group in CHOL. Based on this promising finding, INTS8 could be considered in the development of promising therapies for CHOL. Previous studies proved that CD4 regulatory T cell infiltration is a prominent immunosuppressive characteristic in CHOL^57^. Although Tregs were relatively high in CHOL, they had no association with INTS8.

Although our study provided insights into the relationship between INTS8 and CHOL, there were still some limitations. Due to the smaller occurrence of cases compared with other common cancers, the sample size for IHC involved in cohort validation (especially for the peritumoural tissue samples) was relatively small. In addition, there were relatively few samples with complete clinical information from the TCGA. However, to avoid bias caused by the small sample size, we used data from both the GEO and TCGA databases to confirm the findings, extended the potential functions and mechanisms of INTS8 to pan-cancer research, and discussed the functions of INTS8 in depth. Although the associations between INTS8 expression and MMR genes and DNMTs was shown, the direct mechanisms require further exploration and solid experimental evidence.

## Conclusion

In summary, we observed that increased INTS8 expression can contribute to malignancies and was directly associated with age, grade, and sex in CHOL. Moreover, the pan-cancer analysis revealed that the altered expression of INTS8, which may be mediated by MMR genes and DNA methylation status, might participate in the development of multiple cancer types. In addition, the high INTS8 group displayed an obvious poor prognosis in terms of OS, DSS, and DFI in multiple cancer types. Our results showed the potential of INTS8 as a therapeutic target for CHOL.

## Supplementary Information

Supplementary Figures.

## Abbreviations

AUCs: Areas under the curve
BER: Base excision repair
CCLE: The Cancer Cell Line Encyclopedia
CHOL: Intrahepatic cholangiocarcinoma
CRC: Colorectal cancer
CYP: Cytochrome P450
DEGs: Differentially expressed genes
DNMTs: DNA methyltransferases
DFI: Disease-free interval
DSBR: Double strand break repair
DSS: Disease-specific survival
FDR: False discovery rate
GEO: Gene Expression Omni
GO: Gene Ontology
GSEA: Gene set enrichment analysis
GTEx: The Genotype-Tissue Expression
HCC: Hepatocellular carcinoma
HIBEC: Human normal biliary epithelial cell line
IHC: Immunohistochemistry
INTS8: Integrator complex subunit 8
KEGG: Kyoto Encyclopedia of Genes and Genomes
MCODE: Molecular Complex Detection
MMRs: Mismatch repair genes
OS: Overall survival
RRA: Robust rank aggregation
STRING: Search Tool for the Retrieval of Interacting Genes
TCGA: The cancer genome atlas
TIICs: Tumor-infiltrating immune cells
