# Supplementary material for: INTS8 is a therapeutic target for intrahepatic cholangiocarcinoma via the integration of bioinformatics analysis and experimental validation
Source: Sci Rep. 2021 Dec 8;11:23649. doi: 10.1038/s41598-021-03017-0 (PMC8654853; doi:10.1038/s41598-021-03017-0)
Supplement: Supplementary file 1 — Supplementary Figures. [file 41598_2021_3017_MOESM1_ESM.pdf]

Supplementary Material

1.Supplementary Figures

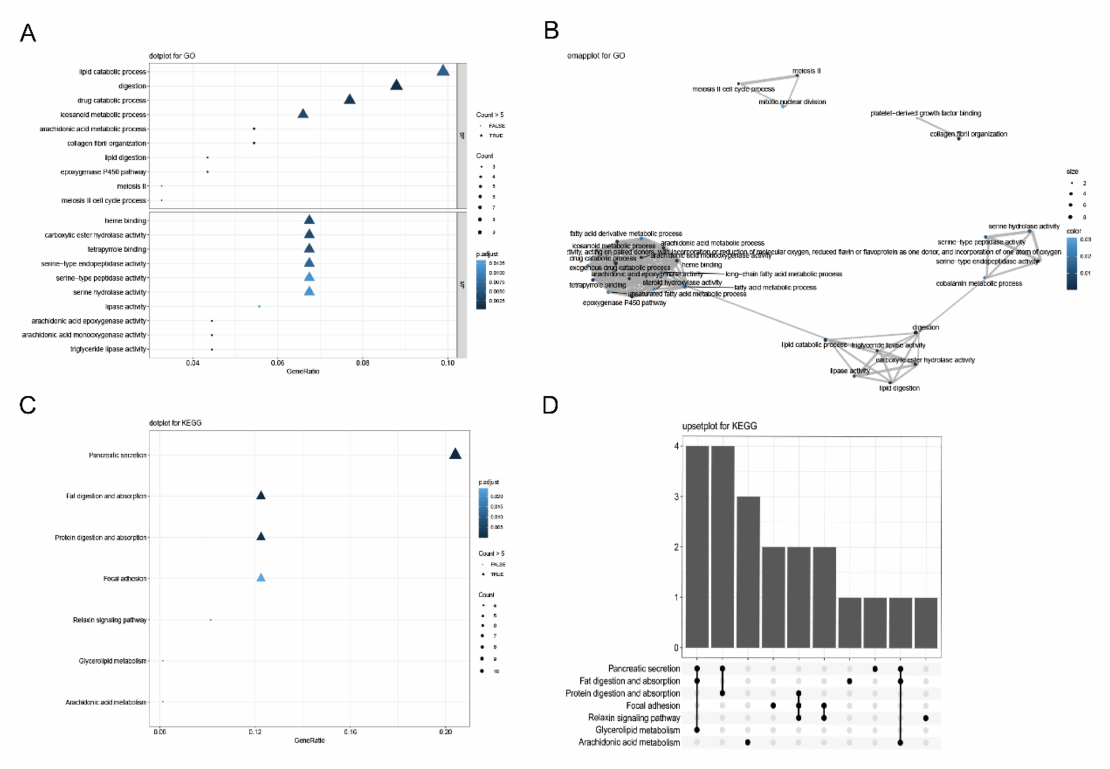

Supplementary Figure 1. Functional enrichment analysis of the RRA gene set. (A-B) GO analysis of RRA gene set. **(C-D)** KEGG analysis of RRA gene set.

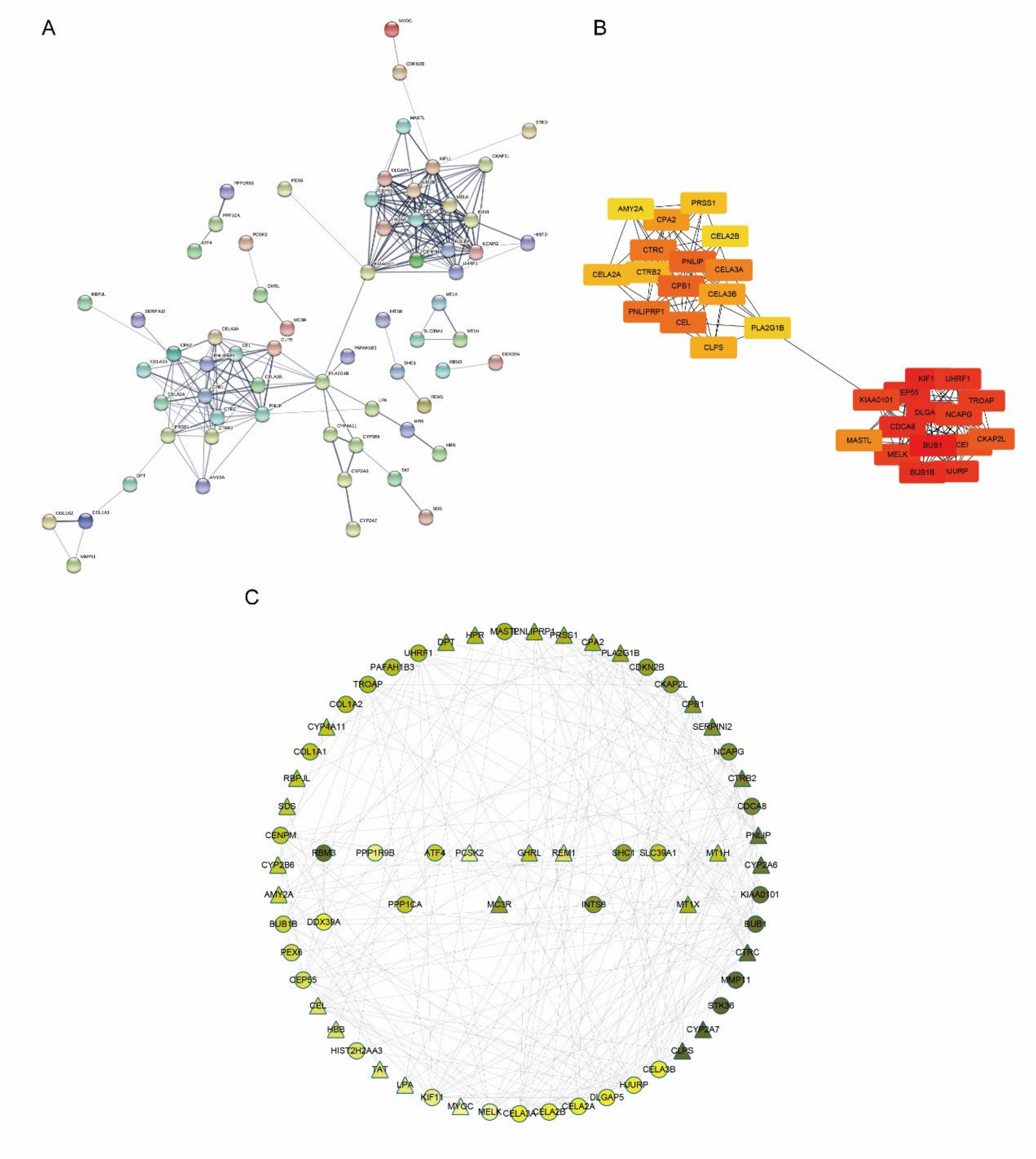

Supplementary Figure 2. PPI network analysis of the RRA gene set. (A) The construction of PPI network by STRING. **(B)** The MCODE model of PPI. **(C)** PPI network visualized by Cytoscape.

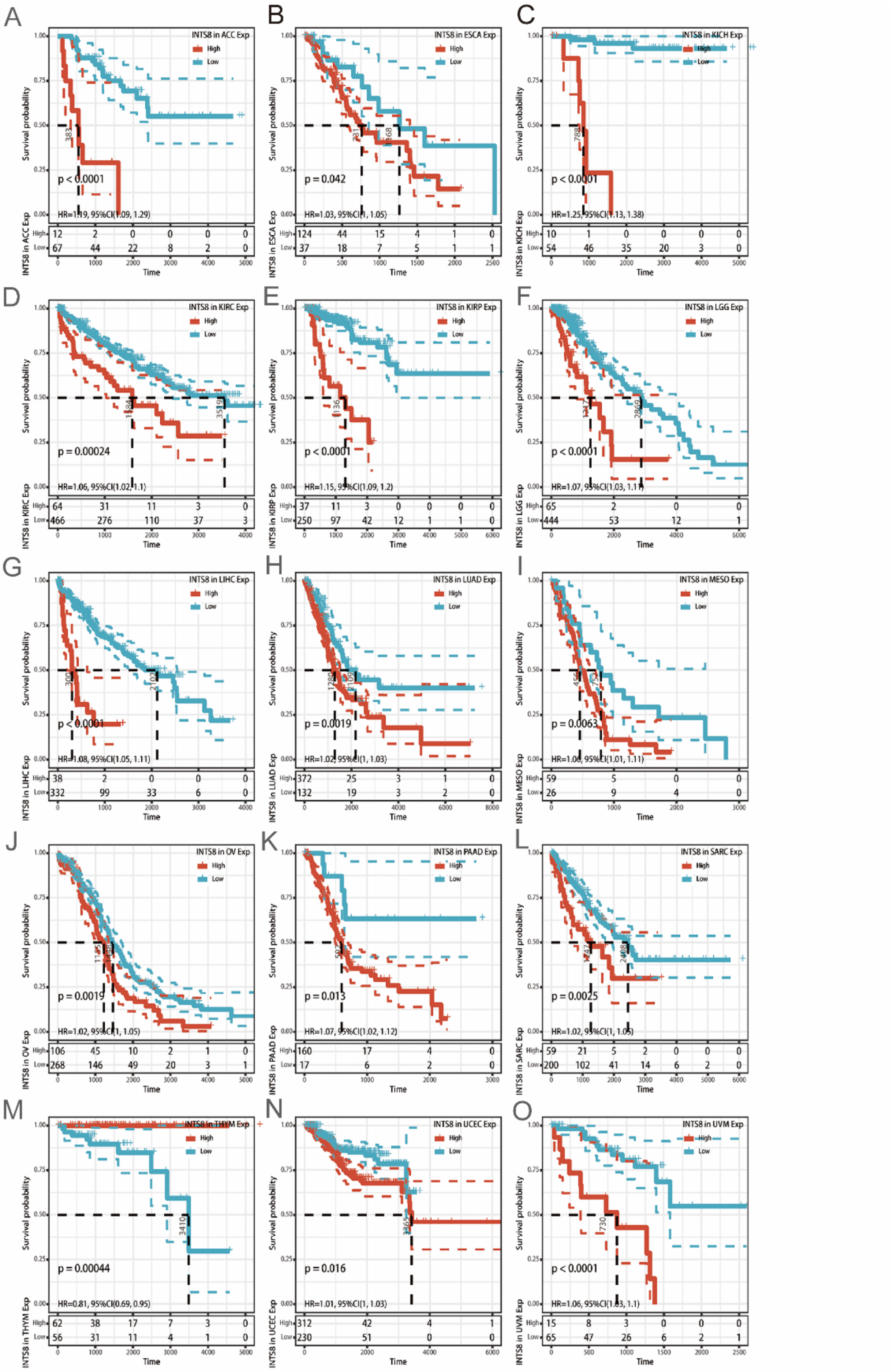

Supplementary Figure 3. The correlation between INTS8 (high and low) and overall survival (OS) in multi-cancer types.

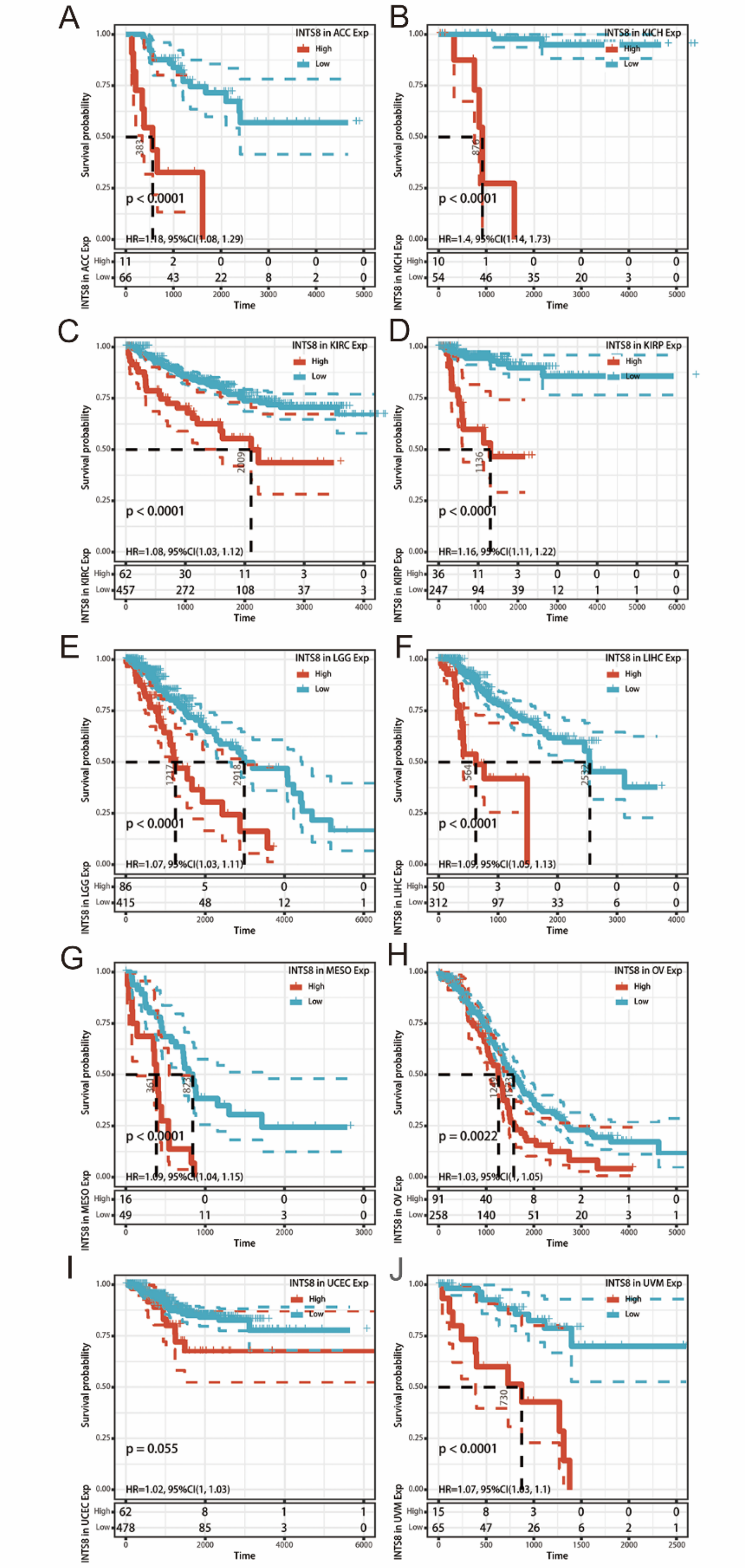

Supplementary Figure 4. The correlation between INTS8 (high and low) and DSS in multi-cancer types.

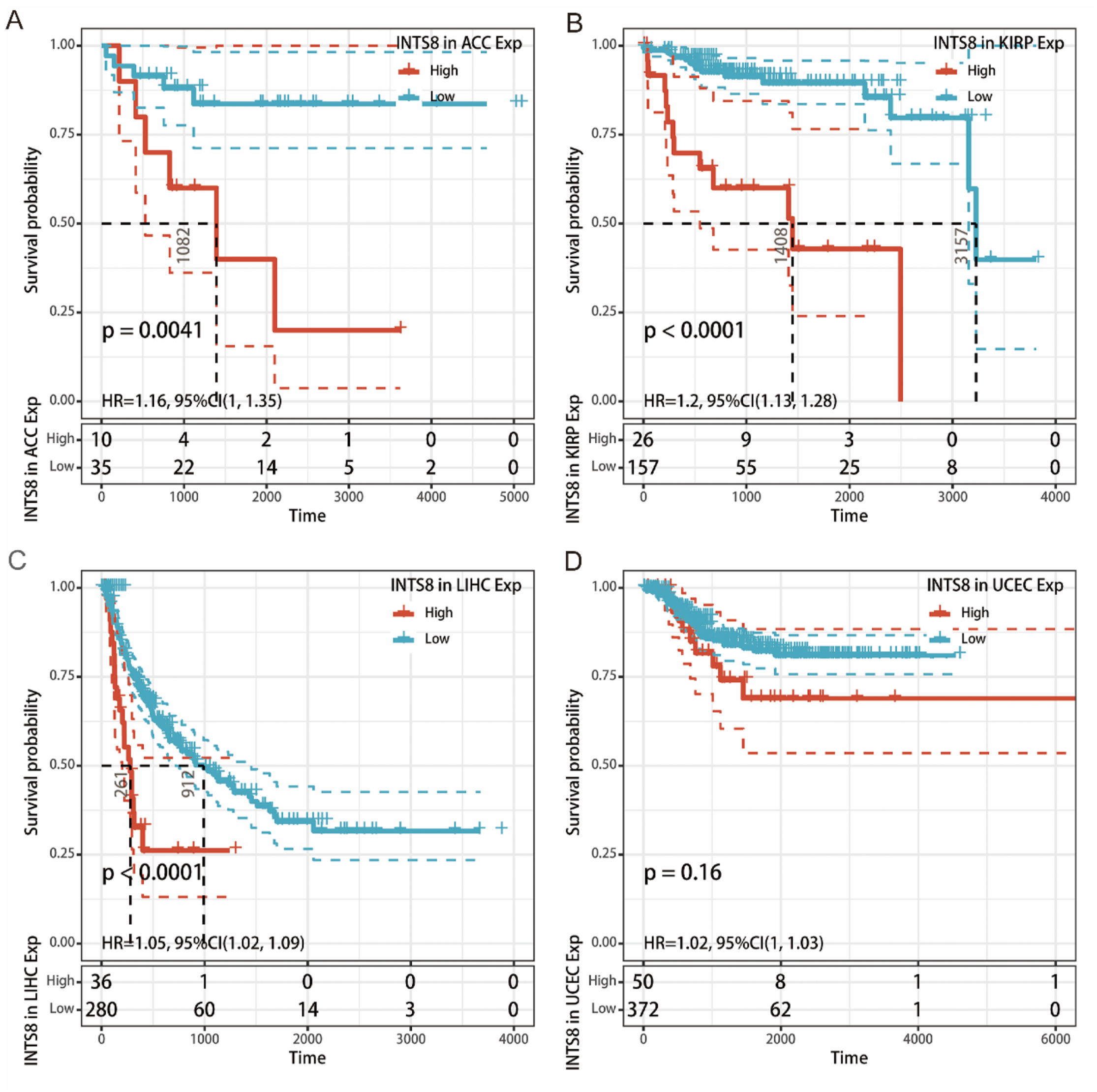

Supplementary Figure 5. The correlation between INTS8 (high and low) and DFI in multi-cancer types.
